# Advancing Bioconjugated Quantum Dots with Click Chemistry and Artificial Intelligence to Image and Treat Glioblastoma

**DOI:** 10.3390/cells15020185

**Published:** 2026-01-19

**Authors:** Pranav Kalaga, Swapan K. Ray

**Affiliations:** Department of Pathology, Microbiology, and Immunology, University of South Carolina School of Medicine, 6439 Garners Ferry Road, Columbia, SC 29209, USA

**Keywords:** glioblastoma (GB), bioconjugated quantum dots (QDs), click chemistry, artificial intelligence (AI), precision imaging, photodynamic therapy (PDT)

## Abstract

Glioblastoma (GB) is one of the most aggressive and invasive cancers. Current treatment protocols for GB include surgical resection, radiotherapy, and chemotherapy with temozolomide. However, despite these treatments, physicians still struggle to effectively image, diagnose, and treat GB. As such, patients frequently experience recurrence of GB, demanding innovative strategies for early detection and effective therapy. Bioconjugated quantum dots (QDs) have emerged as powerful nanoplatforms for precision imaging and targeted drug delivery due to their unique optical properties, tunable size, and surface versatility. Due to their extremely small size, QDs can cross the blood–brain barrier and be used for precision imaging of GB. This review explores the integration of QDs with click chemistry for robust bioconjugation, focusing on artificial intelligence (AI) to advance GB therapy, mechanistic insights into cellular uptake and signaling, and strategies for mitigating toxicity. Click chemistry enables site-specific and stable conjugation of targeting ligands, peptides, and therapeutic agents to QDs, enhancing selectivity and functionalization. Algorithms driven by AI may facilitate predictive modeling, image reconstruction, and personalized treatment planning, optimizing QD design and therapeutic outcomes. We discuss molecular mechanisms underlying interactions of QDs with GB, including receptor-mediated endocytosis and intracellular trafficking, which influence biodistribution and therapeutic efficacy. Use of QDs in photodynamic therapy, which uses reactive oxygen species to induce apoptotic cell death in GB cells, is an innovative therapy that is covered in this review. Finally, this review addresses concerns associated with the toxicity of metal-based QDs and highlights how QDs can be coupled with AI to develop new methods for precision imaging for detecting and treating GB for induction of apoptosis. By converging nanotechnology and computational intelligence, bioconjugated QDs represent a transformative platform for paving a safer path to smarter and more effective clinical interventions of GB.

## 1. Introduction

Glioblastoma (GB) is the most prevalent primary central nervous system tumor that accounts for nearly 50% of all malignant brain tumors [1]. GB has an incidence rate of approximately 0.59 to 5 per 100,000 people internationally, but this number is steadily increasing [2]. Furthermore, the median survival time for adults with GB is between 10 and 14 months for patients receiving radiotherapy, with only 3 to 5% of patients surviving past 3 years and an even lower percentage surviving past 5 years, highlighting the poor prognosis that patients with GB face [3]. Currently, the standard of care for newly diagnosed GB usually follows the Stupp protocol, which is surgical resection followed by radiotherapy and chemotherapy with temozolomide (TMZ) [4]. Once administered, TMZ enters GB cells and acts as a DNA-methylating agent. When replicated, the highly methylated and damaged DNA triggers a DNA danger response in the GB cells, eventually causing cell death [5]. However, while TMZ kills most GB cells, some GB cells adapt by entering senescence, a state that allows GB cells to survive without replication [6]. Once the GB cells leave senescence, they exhibit more invasiveness, tumorigenesis, and resistance to TMZ [7]. Furthermore, recurrence of GB is observed in almost all patients, and the medical community does not seem to have reached a consensus on the standard of care for treating recurrent GB [8]. In addition to its high rates of recurrence, GB presents a distinct challenge for treatment due to its location. One of the greatest difficulties with developing targeted therapies for GB is synthesizing drugs that are small and permeable enough to pass through the blood–brain barrier (BBB) [9]. As such, the challenges associated with the current management of GB require that new therapeutic strategies need to be developed for precision imaging, detecting, and treating GB.

Quantum dots (QDs) are semiconductor nanocrystals with unique optoelectronic properties due to quantum confinement effects that make them ideal for biomedical applications. The most notable features of QDs include size-tunable emission, where the color of the emitted light can be precisely controlled by the particle size, high quantum yield, and exceptional photostability. Smaller QDs emit bluish-to-blue light, while larger ones emit reddish-to-red light. They also exhibit narrow, symmetric emission spectra, which allows for the simultaneous detection of multiple colors with a single excitation source, a property known as multiplexing. Since QDs are resistant to photobleaching, they are highly useful for long-term imaging studies compared to traditional organic dyes [10]. Sizes of QDs can vary usually between 1 and 15 nm, with a wide range of chemical compositions [11]. For example, one group of QDs is group VA, which consists of QDs made from black phosphorus, and another group of QDs is group IV-VI, which consists of QDs made from carbon and silicon compounds [12,13]. The core of a common QD usually has a diameter of 10 to 50 atoms and is composed of approximately 100 to 100,000 atoms in total [14]. Although there are many QDs with varying chemical compositions and structures, they share one property: the ability to fluoresce when exposed to a specific wavelength of light. Furthermore, the same wavelength of light can produce different fluorescent effects depending on the composition of the QDs [15]. Specifically, QDs produce fluorescence through the action of excitons, also known as electron–hole pairs [14]. When excited by a specific wavelength of light, an electron in the QD core valence band jumps to a higher level called the conduction band, leaving behind a positively charged ‘hole’ where the electron used to be in the valence band [16]. Although electrons in the conduction band usually move freely, they are forced to stay within the QD due to its small size and structure in an effect known as zero-dimensional confinement [17]. Unable to leave the QD, the negatively charged electron eventually cools down and returns to its positively charged hole in the valence band, resulting in the release of a photon [14]. Generally, the band gap, which is the amount of energy needed to excite an electron from the valence band to the conduction band in a QD, increases as the size of a QD decreases [18]. This means that smaller QDs shift towards blue emissions while larger QDs shift towards red emissions [19].

QDs have a history of being used as agents for a variety of biomedical applications, such as immunostaining and cancer imaging [10]. Furthermore, through a process called bioconjugation, QDs can be modified to carry ligands that improve QD delivery, biocompatibility, and precision imaging [20]. These bioconjugated or functionalized QDs also show great nanotheranostic potential for simultaneous imaging and treatment of cancers [21]. For the treatment of GB, QDs can be used in coordination with many therapeutics, and one of them is photodynamic therapy (PDT), which uses light to produce reactive oxygen species (ROS) and is a key topic in this review article. The convergence of bioconjugated QDs and artificial intelligence (AI) persuasively offers a powerful new paradigm for improving precision imaging, diagnosis, and treatment of GB. This article explores the synergistic potential of this combined approach, outlining how newly developed bioconjugated QDs provide the necessary precision for imaging and drug delivery, while the emerging AI technology provides the computational power to analyze the resulting data, optimize treatment, and predict GB outcomes. A major focus of this article is to bring together the most recent developments in the use of bioconjugated QDs for precision imaging, diagnosis, and treatment of GB, as well as to illuminate the future directions of the bioconjugated QDs in biomedical applications.

## 2. Challenges with Precision Imaging, Detecting, and Treating GB

One challenge with precision imaging, detecting, and treating GB is pseudo-progression (PsP). PsP is a symptom that shows up in approximately 30% of cases of patients who have received treatments for GB [22]. Although PsP has relatively minimal physical effects on patients aside from increased inflammation and edema, it presents a significant challenge in the identification of GB [23]. If physicians are unable to differentiate PsP from the true progression of GB, it is nearly impossible to measure how effective the treatment is [24]. A possible cause of PsP is the methylation of the promoter of the O6-methylguanine-DNA methyltransferase (MGMT) gene. An earlier clinical study of GB patients receiving TMZ treatment found that 91% of the patients with a methylated promoter in the MGMT gene presented with PsP, while only 41% of patients with an unmethylated promoter in the MGMT gene presented with PsP [25]. However, there is disagreement about this assumption in the earlier clinical observations as other studies have shown inconsistencies between promoter methylation and clinical outcomes [26]. While PsP may not be a symptom with extremely adverse side effects, it presents physicians with the difficulty of differentiating between what is false progression and what is actual progression of GB. This has significant implications for the treatment a GB patient receives.

Another challenge associated with the precision imaging, diagnosis, and treatment of GB is that GB is occasionally misdiagnosed as primary central nervous system lymphoma (PCNSL). While these two cancers are usually differentiable by morphology, there are cases where they present similarly [27]. A common way that PCSNL can be mistaken for GB is if the PCSNL presents with visible necrosis, which is atypical in PCSNL but typical in GB. In fact, visible necrosis is so common in GB that neuropathologists consider it an essential attribute for the undisputable diagnosis of GB in most of the cases. However, this can also appear the other way, as a few GB cases with no visible necrosis presenting as PCSNL [28]. Additionally, while PCSNL can be distinguished from GB by brain biopsy, many patients with either type of tumor are on glucocorticoids such as dexamethasone to control swelling in the brain due to the development of GB and PCSNL. However, the use of glucocorticoids can trigger temporary lysis of PCSNL cells, resulting in an inconclusive biopsy [29]. As such, physicians are put in a tight spot regarding what treatment to administer, considering that the two tumors have vastly different treatment directions. Typical treatment of PCSNL requires either extensive chemotherapy or radiation therapy, while treatment of GB usually involves resection of the tumor and subsequent chemotherapy and radiation therapy [30]. Although PCSNL accounts for only about 5% of all primary brain tumor cases, the possibility that GB is misdiagnosed as PCSNL still presents a distinct challenge in the imaging and treatment of true GB.

A formidable challenge with the treatment of GB is the occurrence of intratumoral heterogeneity (ITH). ITH is a unique mechanism of this tumor evolution that leads to the development of multiple genetically distinct regions in the same tumor mass. These distinct regions highlight the capacity of GB to adapt to its microenvironment, dramatically enhancing the chance of tumor progression [31]. One study of 51 GB samples across 10 patients found strong genetic diversity across the tumor fragments, and even samples from the same patient presented with different genes being amplified, mutated, upregulated, or suppressed. Of the alterations, the genes that are most impacted in GB include mesenchymal–epithelial transition (MET), cyclin-dependent kinase 6 (CDK6), mouse double minute 4 (MDM4), platelet-derived growth factor receptor alpha (PDGFRA), and phosphatase and tensin homolog (PTEN), amongst others [32]. Amplification and/or overexpression of the oncogenes MET [33], CDK6 [34], MDM4 [35], and PDGFRA [36], and mutation or deletion of the tumor suppressor gene PTEN [37], are currently considered the major drivers for the continuous growth and progression of GB. One of the mechanisms by which GB develops ITH is its robust cell-to-cell network and communication [38]. One example of this is a mutation in the epidermal growth factor receptor (EGFR) of GB cells that results in the amplification of EGFR and its function. One study of heterogeneous GB samples, which had both the mutated and wild-type EGFR, found that cells with the mutated EGFR influenced the cells with wild-type EGFR to replicate more and spread via the use of soluble factors like interleukin-6 (IL-6) and hepatocyte growth factor (HGF) [39]. Furthermore, this led to amplification of the Src (short for sarcoma, a non-receptor tyrosine kinase) protein in cells with wild-type EGFR. Amplification of Src protein is a key process in GB proliferation as it effectively reshapes the metabolic system in GB cells to better produce acetyl-CoA and nicotinamide adenine dinucleotide phosphate hydrogen (NADPH) [40]. Due to the extreme genetic variety it produces, ITH poses a huge challenge against the precision treatment of GB.

**Figure 1:**
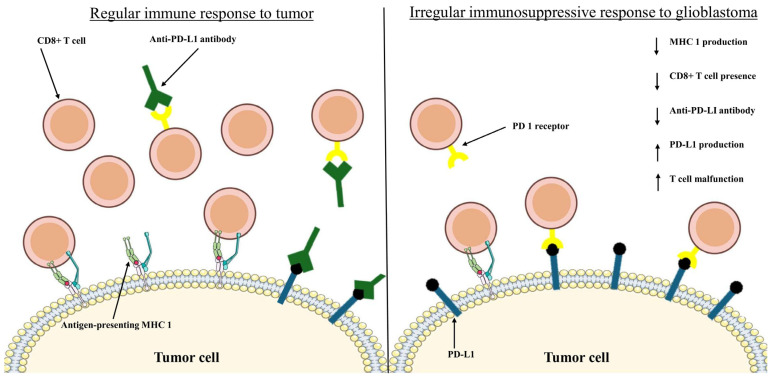
Difference between the normal immune response to a tumor and the immunosuppressive response of GB. There is a lack of CD8+ T cell presence in the immunosuppressive response triggered by GB due to decreased production of MHC1. Furthermore, an increase in programmed cell death protein—ligand 1 (PD-L1) and a decrease in the anti-PD-LI antibody lead to inactivation of any T cells that do make it to the tumor site.

Another challenge with the treatment of GB is its immunosuppressive nature. To survive, GB can promote production of myeloid-derived suppressor cells, regulatory T cells, and IL-10, all of which participate in the inhibition of effector T cells that are responsible for eradicating cancerous cells [41]. Another mechanism through which GB suppresses the immune system is the inhibition of the major histocompatibility complex 1 (MHC1) [42]. Normally, the MHC1 serves as a check against tumorigenesis by forcing tumor cells to present foreign antigens on the cell surface that effector T cells can detect, marking the tumor cells for destruction (Figure 1). However, GB cells have shown the ability to downregulate expression of MHC1, enabling them to evade immune recognition [43].

Despite these challenges, QDs have been shown to reverse the immunosuppressive effects of GB. One study used graphene oxide QDs to reduce the immunosuppressive effects of GB in vivo and conjugated the graphene oxide QDs with resiquimod (R848) and anti-PD-L1 antibody [44]. Conjugation with R848, an immunomodulatory drug, is effective at reducing the immunosuppressive effects of GB as R848 activates Toll-like receptor 7 (TLR7) and TLR8 [45]. Activation of TLR7 and TLR8 is crucial to strengthen the immune response, as this pathway has been shown to increase T cell activity and reduce GB progression [46]. Additionally, conjugation with anti-programmed cell death protein—ligand 1 (PD-L1, a transmembrane protein) antibody inhibits PD-L1, which is overproduced by GB and correlates with significantly poorer patient outcomes [47]. In GB, overproduction of PD-L1 causes significantly increased binding to the programmed death 1 (PD 1) receptor of T cells. As such, when bound with T cells, PD-L1 blocks T cells from marking the cells for targeting by the immune system, enhancing the immunosuppressive effects of GB [48]. After injection with these R848/anti-PD-L1 antibody-conjugated graphene oxide QDs, mice with ALTS1C1 (a type of murine astrocytoma cell line) cells were reported to have decreased tumor size as well as increased the presence of CD4+ and CD8+ T cells at the tumor sites, indicating increased activity of T cells and lowered immunosuppression [44]. In another study, researchers used graphene oxide QDs functionalized with carboxylic acids and TMZ to trigger anti-tumor activity and overcome immune suppression in an in vitro model of GB spheroids [49]. The use of TMZ-conjugated QDs highlights the application of QDs’ targeted drug delivery. Furthermore, this study relied on a method called photodynamic therapy (PDT), which will be described in connection with QDs more thoroughly later in this article. In PDT, select molecules produce ROS when exposed to specific wavelengths of light, and in this study, the conjugated QDs produced ROS when exposed to light at 808 nm [50]. The ROS produced by PDT are crucial to strengthening the immune response to GB, as they have been shown to stimulate production of cytokines, tumor-associated antigens, and damage-associated molecular patterns within the tumor cells that trigger anti-tumor immune activity [51]. The use of bioconjugated QDs as agents for drug delivery, anti-tumor activity, and bioimaging highlights the nanotheranostic potential of QDs, which will also be thoroughly described later in this article.

## 3. Click Chemistry for Producing Bioconjugated QDs for Biomedical Applications

Click chemistry provides an exceptionally efficient and versatile method for creating bioconjugated QDs, which are crucial for use in advanced bioimaging, diagnostics, and targeted drug delivery of different diseases, including cancers [52,53]. By leveraging highly specific and robust reactions, click chemistry overcomes many of the limitations of traditional bioconjugation methods, avoiding poor yield, non-specificity, and the use of harsh conditions that can denature biomolecules or quench the luminescence of the QDs. The approach with click chemistry enables designing stable, covalent attachment of a wide range of biomolecules to QDs, creating powerful and reliable nanoscale probes—bioconjugated QDs—for biomedical applications [54].

Generally, QDs are synthesized in organic solvents and capped with hydrophobic ligands to control their growth and stability. To be useful in biological systems, which are aqueous environments, QDs must be made water-soluble and biocompatible. These desirable attributes are achieved through surface modification of QDs, often by encapsulating them in a hydrophilic polymer or performing a ligand exchange [55,56]. Once the QDs are water-soluble, they require bioconjugation—the process of covalently linking them to biomolecules like antibodies, peptides, or nucleic acids [57]. These biomolecules serve as targeting ligands, directing the QD to specific biological targets, such as a particular protein on a cell surface or a tumor biomarker [58]. However, traditional bioconjugation methods, like carbodiimide coupling, often suffer from low efficiency and non-specific binding, and can negatively affect both the QD’s fluorescence and the biomolecule’s function. This is where click chemistry offers a superior alternative for creating bioconjugated QDs.

Click chemistry was discovered by K. Barry Sharpless and colleagues in 2001 to conduct a set of modular, wide-scope reactions that are high-yielding, rapid, and generate minimal and easily removable byproducts [59,60]. Click chemistry reactions are self-locking due to their large thermodynamic driving force. In the context of bioconjugation, click chemistry reactions are highly specific and bioorthogonal, meaning that they can proceed selectively and efficiently in a biological environment without interfering with other processes in living systems [61].

**Figure 2:**
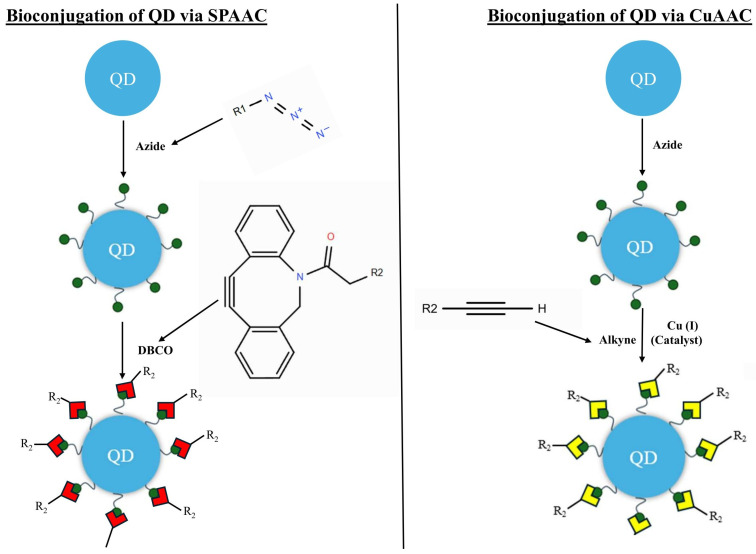
Difference between bioconjugation via SPAAC and CuAAC. In SPAAC, QDs are first conjugated with an azide functionality. Then, they are conjugated with dibenzocyclooctyne, DBCO, which has alkyne functionality. In its R2 group, DBCO can carry a biomolecule for specific targeting. In CuAAC, QDs are first conjugated with an azide functionality. Then, they are conjugated with an alkyne. The reaction between the azide and alkyne is catalyzed by a copper-1 catalyst. The alkyne has a R2 group, which can carry a biomolecule for specific targeting.

The synthesis of bioconjugated QDs using click chemistry usually follows a multi-step process. The process begins with the synthesis of QDs, usually in an organic solvent. The QDs are then modified to be water-soluble and to display a “clickable” functional group on their surface. For example, QDs can be coated with a polymer containing a terminal alkyne or azide group. A common strategy involves encapsulating the hydrophobic QDs in a lipid-polyethylene glycol (PEG) micelle, with the PEG chains terminating in an alkyne or azide handle. This method not only renders the QDs water-soluble and non-toxic but also provides a stable scaffold for bioconjugation [52]. Concurrently, the biomolecules of interest (e.g., antibody, peptide, or DNA) are modified to incorporate the complementary clickable functional group. For example, a protein can react with a molecule containing an azide or alkyne, ensuring that the attachment point does not interfere with its biological activity. The last step is the click reaction itself. The functionalized QDs and the modified biomolecules are mixed under mild, aqueous conditions. The rapid and specific reaction between the azide and alkyne groups, either copper(I)-catalyzed azide-alkyne cycloaddition (CuAAC) or strain-promoted azide-alkyne cycloaddition (SPAAC), leads to the formation of a stable covalent bond, effectively linking the biomolecule to the QD surface (Figure 2) [62].

The use of click chemistry for producing bioconjugated QDs offers several key advantages. The reactions are nearly quantitative, resulting in a high degree of bioconjugation and showing high efficiency and yield. The reactive groups (azide and alkyne) are not found in biological systems, ensuring that the reaction occurs exclusively between the QD and the target biomolecule, providing bioorthogonality. Moreover, the reactions can be performed at room temperature and in aqueous media, preserving the delicate structure and function of the biomolecules and the optical properties of the QDs. A wide range of biomolecules can be conjugated to QDs using this method, making it a universal strategy for creating custom-made probes, ensuring versatility.

Further analyzing Table 1, it becomes clear that biologically relevant ligands dominate for conjugation to QDs: RGD peptides targeting αvβ3 integrins enable highly specific tumor imaging in vitro and in vivo, while guanxgitoxin-1E demonstrates selective labeling of cells with elevated potassium channel expression, underscoring receptors and ion channels as key targets. Regarding composition and safety of QDs, carbon QDs exhibit the most favorable biocompatibility and lowest cytotoxicity profiles, whereas Cd-based QDs provide strong imaging capabilities but depend heavily on surface passivation and ligand choice to mitigate toxicity. Despite these advances, several knowledge gaps remain. We still lack long-term data regarding in vivo toxicity, clearance, and biodegradation, especially for heavy-metal QDs. Direct head-to-head comparisons of ligands and targeting receptors on identical QD cores are scarce, limiting clear identification of optimal design rules. Future research should focus on standardized comparative studies, extended in vivo evaluations, and the development of non-toxic QDs conjugated with disease-specific targeting ligands to accelerate clinical translations. On a brighter note, bioconjugated QDs created via click chemistry have found widespread applications in bioimaging, diagnostics, and therapeutic delivery (Table 1). In bioimaging, they can be used to label and track specific cellular components or to visualize dynamic biological processes in real-time with high resolution and stability. For diagnostics, bioconjugated QDs are used in assays for the sensitive detection of biomarkers, such as in cancer diagnosis [63]. Despite considerable progress, some challenges continue to linger. The long-term in vivo toxicity of heavy-metal-containing QDs (like those based on cadmium or lead) continues to be a major concern, prompting research into less toxic alternatives like graphene QDs or indium phosphide-based QDs [64]. Additionally, optimizing surface chemistry to prevent non-specific interactions and improve QD clearance from the body are active areas of research. As click chemistry continues to evolve with the discovery of new bioorthogonal reactions, it will remain a cornerstone for the creation of next-generation bioconjugated nanomaterials, including QDs.

## 4. Applications of Bioconjugated QDs for Management of GB

### 4.1. Design and Bioconjugation of QDs

**Figure 3:**
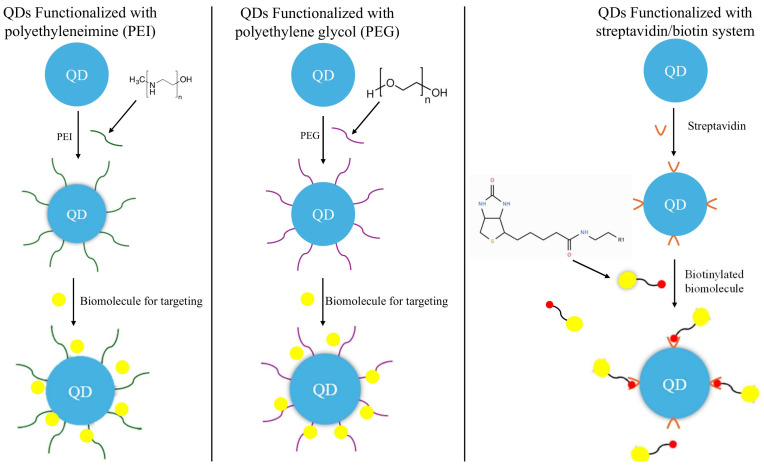
Common ways by which QDs can be functionalized. Three common methods for functionalizing QDs are conjugation with either polyethyleneimine (PEI), or polyethylene glycol (PEG), or the streptavidin/biotin system. These three methods enabled the creation of more biocompatible QDs that carry molecules for specific targeting.

The use of bioconjugated QDs for imaging has already been established in a variety of preclinical (both in vitro and in vivo) studies. One study in mice used QDs modified with dextran to track macrophage analysis. Dextran is a water-soluble polymer that is biologically inert and has high receptor specificity [72]. After adding dextran to the outer shell of (CdSe)-ZnS core/shell QDs, researchers noted longer circulation time and improved brightness of macrophage imaging from fluorescence microscopy compared to dextran controls in mice models [73]. Furthermore, a study with dextran-conjugated QDs focused on developing super-QDs. These super-QDs are a collection of red-, blue-, and green-emitting QDs combined into one exceptionally large particle that fluoresces extremely bright [74]. Researchers were even able to image these super-QDs in vitro through a smartphone camera, highlighting the potential ease and accessibility for patients and providers that can come with the development of QD-based imaging [75]. There are several methods that are currently used for functionalizing the QDs (Figure 3). One study used polyethyleneimine (PEI)-modified silicon QDs to deliver DNA in vitro [76]. PEI is used to enhance delivery outcomes by serving as a hydrophilic skeleton and providing the QDs with a positive charge [77]. In the study, researchers used PEI to cover the silicon QDs with a hydrophilic coating that would bind DNA. From there, the PEI/DNA-silicon QDs were encapsulated with cell membrane from red blood cells to protect the QDs and reduce any cytotoxic effects. Researchers found that the membrane encapsulated PEI/DNA-silicon QDs showed higher transfection rates of 293T cells and HeLa cells than silicon QDs that were directly bound with DNA [76]. One study in human plasma serum used (CdSe)-ZnS core/shell QDs conjugated with NS1 antibodies and streptavidin to measure the presence of the NS1 antigen, a marker of dengue fever [78]. Using these bioconjugated QDs, researchers were able to detect NS1 antigen between 0.001 nM and 120 nM, which appeared significant for early detection of dengue fever. During this type of development, the QDs are first conjugated with streptavidin, which is known for its affinity to biotin. Furthermore, researchers conjugated the NS1 antibody with biotin, known for its ability to improve delivery outcomes as well as fluorescence levels compared to non-biotinylated molecules [79]. The use of streptavidin and biotinylated NS1 antibody ensured that the NS1 antibody securely attached to the QDs and opened an avenue for the development of bioconjugated QDs through the streptavidin/biotin system. Another study used the streptavidin/biotin system to develop bioconjugated QDs that could detect the amount of Salmonella in milk. Researchers created biotinylated Salmonella monoclonal antibodies that could attach to (CdSe)-ZnS core/shell QDs due to the streptavidin on the surface of QDs. Results showed that the bioconjugated QDs displayed significantly higher binding to Salmonella compared to other binding with non-Salmonella bacteria, indicating high specificity. Furthermore, the conjugated QDs were able to detect as little as 4.9 × 10^3^ colony forming units (CFUs)/mL within one hour, highlighting the potential speed and accuracy of this application [80].

Although the bioconjugation of QDs results in a plethora of diverse applications for QDs, there needs to be a better understanding of how these QDs are created to ensure safe and efficient applications. The majority of QDs are developed and transmitted in organic solvents and, likewise, present as hydrophobic [81]. This hydrophobic nature can enable better uptake of QDs through the non-polar cell membrane [82]. However, for QDs to be more biocompatible, they need to be hydrophilic, which would promote greater stability in an aqueous environment [83]. There are three ways by which hydrophilic QDs can be synthesized: creation in aqueous solutions, replacing the hydrophobic surface ligands in the shell with hydrophilic surface ligands, and creating a hydrophilic shell to cover the main hydrophobic shell [84]. Although the synthesis of QDs in aqueous solutions was once considered a laborious and low-yielding method, the development of hydrothermal synthesis has made the creation of biocompatible QDs in aqueous solutions more feasible and high-yielding. Hydrothermal synthesis utilizes extreme pressure and temperature to crystallize the target product from a water solvent [85]. One study measuring the effect of temperature on the production of QDs found that SnO_2_ QDs grown at 125 °C were, on average, 1.3 nm smaller than SnO_2_ QDs grown at 225 °C [86]. Moreover, temperature and pressure are not the only conditions that can be modified to change QD growth outcomes. Researchers in another study using hydrothermal synthesis to grow QDs utilized different reducing agents and modified pHs to study CdSe QD production. They found that CdSe QDs that were grown in an aqueous solution with a pH of 9 in the presence of the reducing agent sodium borohydride produced the strongest emissions [87]. In a study on graphene QDs, researchers were able to control the quantity and presence of various oxygen-based surface ligands through hydrothermal synthesis. Graphene QDs synthesized under acidic conditions and higher temperatures presented with more hydroxyl groups than carboxyl groups, while graphene QDs synthesized under basic conditions and lower temperatures presented with more carboxyl groups than hydroxyl groups. The groups of graphene QDs that showed the greatest quantum yield and photoluminescent intensity were the ones synthesized under basic conditions and elevated temperature [88]. This study highlights how hydrothermal synthesis can also impact surface ligands of QDs, which are crucial to the biocompatibility of QDs. This is especially relevant to the development of biocompatible QDs, as another strategy to make QDs hydrophilic through the attachment of functionalized surface ligands.

### 4.2. Development of Amphiphilic Bioconjugated QDs

Surface ligand specificity is crucial to QD performance, and improper surface ligand–host tissue interactions can trigger toxic effects [89]. One study in *Caenorhabditis elegans* found that CdSe/ZnS QDs coated with the hydrophobic molecule trioctylphosphine oxide caused significantly more toxic effects in *C. elegans* than the CdSe/ZnS QDs coated with hydrophilic molecules such as dihydrolipoic acid and polyethylene glycol (PEG) [81]. Another study with CdSe/ZnS QDs focused on targeting GB in mice models by modifying the QDs with the ligands, PEG, and aptamer 32 (A32). A32 is a single-stranded DNA sequence that shows a selective affinity for EGFRvIII, a mutated growth factor receptor present in about 30% of GB cases [90]. Using the streptavidin/biotin system discussed earlier, researchers were able to attach A32 to the surface of PEGylated CdSe/ZnS QDs and specifically bind GB cells in mice models. Furthermore, researchers tracked mice weight and analyzed postmortem tissue samples from across the body to identify any toxicity but were unable to find any changes suggestive of the QD triggering no toxic effects in the mice [91]. Although the functionalization of QDs for biocompatibility with surface ligands is promising, there needs to be a thorough understanding of how the functionalized ligands interact with the host environment. The third strategy for the development of biocompatible QDs is encapsulating the QD with a water-soluble shell. Although a promising technique, the water-soluble shell that covers the QD must not inhibit the fluorescence of the QD. One molecule that shows promise as the foundation for a secondary water-soluble shell is siloxane [92]. One study with siloxane-coated QDs found that coated QDs were able to resist environmental degradation from exposure to sodium hydroxide, hydrochloric acid, and ethanol. The coated QDs also functioned normally while tested under temperatures of up to 85 °C and 85% relative humidity [93]. Another study tested these siloxane-coated QDs in boiling water and found that these coated-QDs displayed enhanced photoluminescent emission despite the extreme temperature. Researchers attributed this resistance against the extreme conditions to the siloxane coating, which passivates surface defects known as trap states in QDs [94]. The strategy of developing hydrophilic shells around the hydrophobic cores of QDs gives way to the creation of amphiphilic QDs that show great promise as biocompatible agents for imaging.

The future of biocompatible QDs for precision imaging rests heavily upon advancements in the development of amphiphilic QDs [95]. However, the development of amphiphilic QDs has its own set of issues. One of the biggest problems associated with amphiphilic QDs is that the sizes of these QDs become way too large due to the extensive coating of their surface with amphiphilic polymers. It is estimated that coating with amphiphilic polymers can increase QD size to almost double the size of QDs created via surface ligand exchange [96]. However, advancements in QD development have generated amphiphilic QDs with sizes smaller than QDs developed through surface ligand exchange. One study focused on the creation of silicon QDs for oil recovery developed silicon QDs coated with an amphiphilic mixture of 2-ethylhexyl glycidyl ether and various water-soluble groups. The resulting QDs were only 3.5 nm, which shows how far the development of amphiphilic QDs has come [97]. Although this study created amphiphilic silicon QDs for oil detection, further study using this technique to create amphiphilic silicon QDs for in vitro and in vivo imaging studies would be useful, since silicon QDs are non-toxic and demonstrate great biocompatibility [98]. Another challenge with creating amphiphilic QDs is that both surface ligand exchange and encapsulation with amphiphilic polymers can impact the production of the amount of evenly sized, or monodisperse, QDs in a solution [99]. However, one study was able to tackle this issue using grafting, which is the process of attaching polymers to other molecules. In this study, researchers combined octylamine chains with carboxyl groups on polyacrylic acid to create a larger amide polymer, forming an extensive amphiphilic coating on the surface of CdSe/CdS QDs. Moreover, researchers noted that the grafting ratio, the number of carboxyl groups converted to an amide, was key to controlling size distribution. For example, a QD with a 5 nm diameter and a grafting ratio of 42 would result in QDs with a uniform size distribution, but a QD with an 8 nm diameter and the same grafting ratio of 42 would result in a much more inconsistent size distribution. This suggests that when grafting, the grafting ratio needs to increase as QD size increases to create QDs with uniform size distribution [99]. Although this method is promising, one downside is that it creates QDs with a large size due to the extensive surface coating [96]. This increased size can present problems for imaging as larger QDs have a greater affinity for non-specific binding as well as greater trouble navigating crowded and narrow areas like a neuronal synapse [100]. One way to resolve the size issue associated with grafting amphiphilic polymers is through using a molecule that combines an amphiphilic polymer with a zwitterion, a molecule that has both a positive and negative charge [101]. In one study, researchers used the octylamine-grafted polyacrylic acid mentioned in the prior study but modified it with pyridinium propane-1-sulfonate, a zwitterion. Furthermore, researchers tested this new amphiphilic zwitterionic coating on four different QDs and found that all QDs with an amphiphilic zwitterionic coating were significantly smaller than their counterparts with an octylamine-grafted polyacrylic acid coating [96]. Advancements in amphiphilic QDs present a fantastic opportunity in the creation of biocompatible QDs, and further developments in the synthesis of amphiphilic QDs are crucial for the use of QDs as imaging agents for GB.

### 4.3. Bioconjugated QDs for Precision Imaging, Detecting, and Treating GB

Although GB presents a tough challenge for imaging and diagnosis, QDs have shown their capability as agents for the targeted precision imaging and detecting various cancers, including GB. In one study, researchers bioconjugated CdTe/ZnS QDs with arginylglycylaspartic acid (RGD) peptide to target the U87MG tumor in mice for specific imaging [102]. RGD is a peptide sequence that has been frequently used to target GB cells due to its affinity for the αvβ3 integrin receptor [103]. The αvβ3 integrins have been implicated in promoting the growth and invasion of GB through metabolic reprogramming and exosome propagation [104]. After injection of the bioconjugated QDs with RGD, researchers were able to image the tumor with clear margins and even track tumor angiogenesis in the mice [102]. The specificity of these bioconjugated QD probes demonstrates how QDs can be used to produce images with accurate margins and differentiate GB from PsP and PCSNL. Furthermore, QDs are great tools for the imaging and diagnosis of GB due to their functionality with computed tomography (CT), magnetic resonance imaging (MRI), and positron emission tomography (PET) scans. One study highlighting the functionality of bioconjugated QDs with CT used Ag_2_Te QDs conjugated with bovine serum albumin (BSA) as the contrast agents to image intestinal obstructions in Kunming mice [105]. Because Kunming mice are an outbred strain of laboratory mice originating in China from Swiss mice, they are not genetically uniform, but they are a common model organism due to affordability and suitability for studying diverse neurological disorders, cancers, and drug delivery. Using Kunming mice, researchers noted no significant histopathological damage to the mice after injection of the bioconjugated Ag_2_Te QDs with BSA and were able to use the scans from these QDs for image-guided obstruction surgery [105]. Aptamers are short, single-stranded DNA or RNA molecules, although some are peptides. They can fold into specific three-dimensional shapes for binding with high affinity and specificity to their targets, such as proteins, small molecules, and even cells. A study evaluating the CT functionality of QDs used the bioconjugated Au QDs with AS1411 aptamer, a guanosine-rich oligonucleotide aptamer that shows high affinity for receptors specifically overexpressed in lung cancer models [106]. Researchers then injected these bioconjugated Au QDs with AS1411 into CL1-5, a lung cancer cell line, tumor-bearing mice, and used the resulting CT scans for image-guided surgical resection of the tumors [106]. One study assessing the MRI functionality of QDs has used carbon QDs (CDs) conjugated with gadolinium, a metal ion with paramagnetic qualities, and folic acid, which binds to folic acid receptors that are overexpressed in liver cancer [107]. After their injection in BALB/c mice carrying hepatoma, researchers were able to obtain the targeted MRI scans and noted no significant tissue damage or toxicity within the mice [107]. In another study, researchers developed metal-free QDs for MRI by conjugating graphene QDs with boron [108]. Researchers were able to maintain these MRI images for more than an hour and produce clear images of the heart, liver, lung, kidney, and spleen [108]. Another study focused on metal-free QDs for MRI imaging used conjugated graphene QDs for image-guided surgical resection of tumors in mice bearing the MCF-7 tumor, which is a breast cancer [109]. One study examining the functionality of QD with PET scans used InP/ZnSe/ZnS core/shell QDs chelated with ^64^Cu to image mice bearing A431 tumors, an epidermoid carcinoma [110]. Researchers were able to use these QDs as probes for simultaneous PET scan and fluorescence imaging capabilities, and postmortem analysis of the mice revealed high accumulation of the chelated QDs at the tumor site as well as a lack of abnormal developments in the mice in the month after injection [110]. Through conjugation with specific molecules, QDs can also be potent tools for the imaging and diagnosis of GB. Furthermore, QDs that show functionality with the aforementioned imaging tools can enable accurate image-guided tumor resection, highlighting the versatile potential of QDs to simultaneously image, diagnose, and treat GB.

There have been many interesting studies showing both in vitro and in vivo uses of QDs for precision imaging, diagnosis, and treatment of GB (Table 2). These studies highlighted the design and functionalization of the bioconjugated QDs for use in the preclinical (in vitro and in vivo) models of GB for the purpose of imaging or/and treatment or image-guided surgical resection. The results from these studies revealed some interesting and desirable attributes of bioconjugated QDs, such as their uptake by the GB cells, penetrability through the BBB in GB animal models, specificity and efficacy in inducing tumor cell death, and good renal clearance and lack of side effects in GB animal models. Moreover, these investigations described the molecular mechanisms for the successful imaging and treating of GB in vitro or in vivo using the bioconjugated QDs.

The studies (Table 2) show that biologically derived targeting ligands such as peptides, antibodies, and endogenous ligands are the most effective for achieving high specificity toward GB cells. Among these, RGD-based peptides and folic acid recur most frequently, highlighting αvβ3 integrin and folate receptor-α as dominant and reliable targeting receptors in GB models. Other receptors, such as EGFRvIII, IL13Rα2, CD133, p32, and transferrin receptors, further emphasize that receptor overexpression in GB is central to successful QD targeting and BBB penetration. However, there are some knowledge gaps that remain before broader translation of this concept. Long-term in vivo toxicity, biodegradation, and fate of QDs in the brain are still insufficiently characterized, particularly after repeated dosing. Direct comparative studies across ligands and QD cores under identical conditions are rare, limiting clear structure–function relationships. Future studies should focus on standardized comparisons, extended safety and clearance studies, and the development of heavy-metal-free, multifunctional QDs that can integrate imaging and therapy while maintaining precise receptor-level targeting in GB.

Bioconjugated QDs, with their unique optical properties and versatile surface chemistry, are being developed as a multifunctional platform, not only to overcome the BBB in GB treatment but also to functionalize QDs with targeting ligands and therapeutic agents to combine both the diagnostic imaging and therapeutic functions in a single platform. Another significant area of progress is the development of QDs with enhanced biocompatibility and reduced toxicity. Traditional cadmium-based QDs come with concerns about their potential long-term toxicity. To address this, researchers are focusing on the synthesis of cadmium-free QDs, such as those based on carbon, graphene, and ternary or quaternary semiconductors [125,126]. These newer QDs offer comparable optical properties while being more cytocompatible. Furthermore, the surface of these QDs is readily functionalized with biocompatible polymers and ligands, such as carboxymethylcellulose, to improve their stability in physiological environments and further reduce toxicity in GB therapy [127]. For imaging, bioconjugated QDs have superior photostability, high quantum yield, and tunable emission spectra, all of which offer significant advantages over traditional organic dyes. Recent studies have focused on developing bioconjugated QDs that can specifically target GB cells. For example, QDs have been conjugated with ligands that bind to receptors overexpressed on GB cells [64]. This targeted approach allows for the precise visualization of tumor margins during surgery, which is critical for maximizing tumor resection while preserving healthy brain tissue. In therapeutic applications, bioconjugated QDs are being developed for a variety of treatment modalities, including drug delivery, photothermal therapy (PTT), and PDT [128]. One of the most promising approaches is conjugating QDs with chemotherapeutic drugs such as doxorubicin or TMZ for treating GB. These QDs function as nanocarriers, facilitating targeted delivery of the drug to the tumor cells and reducing potential systemic toxicity.

The development of bioconjugated QDs thus represents a transformative advancement in the management of GB. From enhancing surgical precision through multiplexed bioimaging to enabling targeted drug delivery and synergistic phototherapies, these multifunctional nanoplatforms hold immense promise for the management of GB. While challenges related to long-term toxicity and clinical translation remain, the continuous evolution of QDs and bioconjugation strategies is paving the way for more effective, personalized, and less toxic treatment options for patients with this deadly brain tumor.

## 5. Improving BBB Permeability and Intracellular Intake Mechanisms of Bioconjugated QDs

One of the most important factors impacting the efficacy of QDs to image, diagnosis, and treat is their ability to cross the BBB and reach the GB tumor site. While QDs are reported to cross the BBB, this capability is not intrinsic to all QDs and depends strongly on their physicochemical properties, particularly size, surface chemistry, and composition [129]. Current evidence supports distinct dominant transport mechanisms for different QD classes, rather than a universal BBB-crossing behavior. Of note, there are three major QD classes with unique methods to cross the BBB: amphiphilic QDs, larger QDs that lack amphiphilicity, and carbon QDs. Amphiphilic QDs, specifically those characterized by a small hydrodynamic diameter and balanced hydrophilic–lipophilic surface chemistry, have been proposed to cross the BBB primarily through diffusion-based mechanisms. In one study on zebrafish, researchers used amphiphilic CDs to successfully cross the BBB. Researchers were confident that diffusion was the primary mechanism of crossing the BBB since the CDs were less than 5 nm in size. Furthermore, researchers repeated this experiment with similarly sized hydrophilic CDs and were unable to detect significant BBB permeability, highlighting the importance of amphiphilicity in crossing the BBB [130]. Importantly, “diffusion” in this context should not be interpreted as classical free diffusion through intact tight junctions. Instead, amphiphilic QDs may exploit transcellular diffusion-like processes, involving passive partitioning into endothelial cell membranes followed by slow traversal across the cell body. This mechanism is most plausible for ultrasmall QDs that are typically less than 5 to 6 nm hydrodynamic in diameter, where membrane insertion and vesicle-independent transport become energetically feasible.

Inorganic QDs composed of metal-based cores generally exhibit poor intrinsic BBB permeability due to their larger hydrodynamic size and lack of amphiphilicity. As a result, these QDs rely predominantly on receptor-mediated transcytosis (RMT) to achieve BBB crossing. RMT involves the binding of surface-functionalized QDs to specific receptors expressed on brain microvascular endothelial cells, followed by vesicular internalization and transport across the endothelial layer [131]. Commonly targeted receptors include the transferrin receptor (TfR), insulin receptor, and low-density lipoprotein receptor-related protein 1 (LRP1) [132]. Inorganic QDs functionalized with ligands such as transferrin, lactoferrin, angiopep-2, or specific antibodies have demonstrated enhanced uptake and measurable brain accumulation in vivo. In one study with rats, researchers conjugated CdSe QDs with transthyretin, a protein with a receptor that shows activity across the BBB. The transthyretin-conjugated QDs showed significantly better permeability and uptake in rat brains compared to unconjugated QDs and QDs conjugated with proteins that did not have receptors with activity across the BBB [133]. CDs and graphene QDs represent a distinct class with physicochemical properties more closely resembling small organic molecules than classical nanoparticles. Notably, CDs and other nanoparticles, especially those derived from glucose, have been shown to cross the BBB via carrier-assisted transport, particularly through glucose transporters (GLUTs). GLUT1 is abundantly expressed on BBB endothelial cells and is responsible for maintaining cerebral glucose supply [134]. In one study, researchers used CDs derived from glucose to cross the BBB in both rats and zebrafish. Additionally, researchers introduced these glucose-derived CDs to budding yeast that was modified to lack GLUTs and found that the yeast cells were unable to take up the CDs. The study highlights that the inhibition of GLUT1 significantly reduces the uptake of glucose-derived CDs, supporting a GLUT-mediated transport mechanism for glucose-derived CDs [135].

Another key area that affects the capacity of QDs to image GB is their ability to cross the cell membrane and safely enter the cell [136]. The three pathways that are the most involved in QD intake are clathrin-mediated endocytosis (CME), caveolae-mediated endocytosis (CVME), and macro-pinocytosis (MP) [137]. In CME, adaptor proteins on the cell membrane bind select extracellular molecules. Then, clathrin binds the adaptor proteins and forms a circular pit towards the inside of the cell. Eventually, this pit is disconnected from the cell membrane by dynamin proteins and brought into the cell, allowing the chosen extracellular molecules to enter the cell [138]. One study used QDs as agents of drug delivery to deliver S15-APT, an aptamer coded against specific lung cancers, to A459 cells with non-small cell lung cancer in vitro. Researchers found that this modified QD was only able to enter A459 cells despite also being evaluated against healthy bronchial epithelial BEAS2B cells, cervical carcinoma HeLa cells, and colon adenocarcinoma CaCo-2 cells, suggesting that the CME pathway shows high specificity for QDs with target ligands [139]. Another study using QDs for single-virus tracking found that Influenza A-coupled QDs entered Madin–Darby canine kidney (MDCK) cells and HeLa cells through clathrin-mediated endocytosis. Furthermore, researchers were able to track the QD-labeled Influenza A virus movements along intracellular microtubules and actin filaments, highlighting the use of QDs as imaging agents that can provide dynamic real-time data [140]. The second mechanism of QD uptake is CVME, which follows a similar pathway to CME. However, in CVME, extracellular molecules enter pores in the cell membrane called caveolae and form a pit by binding to caveolin. The pit is then closed off by dynamin proteins, and the molecules enter the cell [141]. One study in the ovarian cancer cell lines SKOV3 and OVCAR3 used CdTe QDs to image the cancerous cells. Researchers observed that the presence of nystatin, a CVME inhibitor, significantly reduced QD uptake compared to cells treated with chlorpromazine, a CME inhibitor. This suggests that CVME is a key mechanism for uptake of CdTe QDs in ovarian cancer cell lines [142]. Although CVME and CME seem to be remarkably similar processes of cellular intake, one major distinction between the two is pit size. The pits formed by CME have an average diameter of 100 nm, while the pits formed by CVME have an average diameter of 60 nm [143]. This highlights how important controlling QD size during development is to ensure successful delivery, since certain cell lines appear to use different size-dependent pathways to intake QDs.

The third pathway commonly used for QD intake is MP, which does not rely on dynamin proteins. Rather, actin fibers at the cell membrane extend from the cell membrane to form “membrane ruffles” that encircle and trap molecules in macropinosomes, large cellular vesicles (0.2 to 5 μm) formed because of macropinocytosis [144]. One study that observed the use of MP to intake QDs focused on analyzing how SDots, the sulfur QDs, conjugated with the Tat peptide, would be brought in by HeLa cells. Researchers found that the HeLa cells used MP to intake the conjugated SDots since the presence of cytochalasin D, an inhibitor of actin polymerization, significantly reduced the fluorescent intensity of HeLa cells [145]. The discovery that HeLa cells use MP to intake conjugated SDots also presents evidence suggesting that the specific pathway used to intake QDs is dependent on the hydrophilicity of QDs, in addition to their size. The fact that HeLa cells used MP to bring in SDots seemingly demonstrated poor hydrophilicity despite being conjugated [145,146]. However, in the previously mentioned study using Influenza A-coupled QDs, HeLa cells used the CME pathway to bring in QDs. The QDs in this study were conjugated via the streptavidin/biotin system and were coated with an external polymer shell [140]. The modification of these QDs with the streptavidin/biotin system and external polymer shell would confer these QDs with strong hydrophilic properties [147]. The difference in pathways that HeLa cells used to intake QDs with opposing measures of hydrophilicity suggests that this cell line can modulate the endocytic pathway it uses depending on the hydrophilicity of the chosen QD. A thorough understanding of how modifying variables like QD size and hydrophilicity can impact the pathway by which QDs enter cells would greatly benefit the efficient delivery of QDs to GB.

CME, CVME, and MP are three forms of endocytosis that are energy-dependent. However, QDs have been shown to diffuse through the cell membrane if the QDs are amphiphilic [148]. One way to track the diffusion of QDs across the cell membrane is by using giant plasma membrane vesicles (GPMVs), which are vesicles that model the structure of the cell membrane but lack the ability to use any endocytic pathways [149]. One study used GPMVs to track the movement of amphiphilic CdTe QDs across the cell membrane. Researchers confirmed that amphiphilic CdTe QDs were able to enter the GPMVs despite the lack of any endocytic pathways. In the same study, researchers also compared the pathways by which amphiphilic and hydrophilic CdTe QDs enter CHO cells. They found that CHO cells that were exposed to hydrophilic CdTe QDs formed endosomes containing hydrophilic QDs, indicating that the CHO cells used an endocytic pathway to bring in the hydrophilic QDs. However, in CHO cells that were exposed to the amphiphilic CdTe QDs, researchers were unable to find any endosomes containing the QDs and observed a dispersed distribution of QDs in the cytoplasm, suggesting that the amphiphilic QDs were able to pass through the cell membrane without any endocytic pathway [150]. Another study interested in the cellular mechanisms of the intake of amphiphilic QDs analyzed the pathways by which modified CDs entered HeLa cells. The amphiphilic QDs were made with hydrophilic cellulose and ascorbic acid coating, and the addition of lipophilic capsanthin tails. The amphiphilic CDs were introduced to HeLa cells that had been exposed to various endocytic inhibitors like nystatin and cytochalasin D, and despite the presence of these inhibitors, the amphiphilic CDs were still able enter the cell, suggesting they could cross the cell membrane without endocytosis. Moreover, researchers repeated this experiment with CDs that had their capsanthin tails removed and found that the modified CDs were unable to enter HeLa cells in the presence of endocytic inhibitors, boosting the evidence that amphiphilic QDs could pass through the cell without energy-dependent endocytic pathways [151]. Additional research into the pathways that specific GB cell lines use to intake QDs, whether energy-dependent or not, will greatly benefit the development of QDs that effectively target and reach GB.

## 6. Bioconjugated QDs in Conjunction with PDT for Treatment of GB

**Figure 4:**
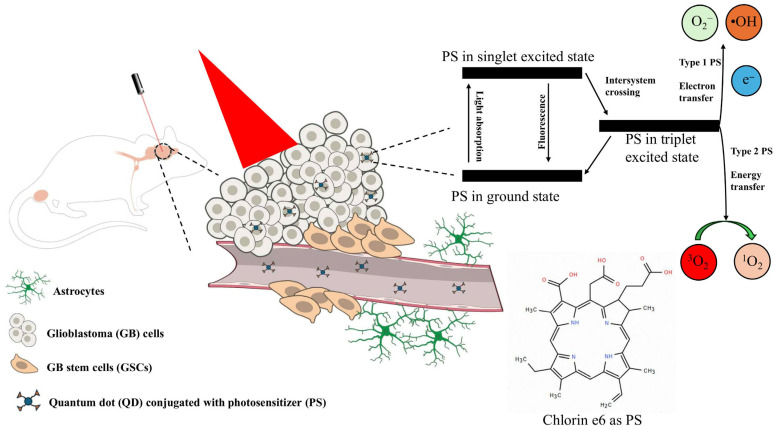
Basic mechanism of QDs and PDT for treating GB. QDs carrying PSs cross the BBB and enter the bloodstream, eventually reaching the GB tumor site. Once exposed to a specific wavelength of light, the PSs are activated and start ROS production. Depending on the type of PS, it can either follow the type 1 pathway, which produces superoxide anions (O_2_^−^) and hydroxyl radical (•OH) from electron transfer, or the type 2 pathway, which uses energy transfer to convert triplet molecular oxygen (^3^O_2_) into singlet molecular oxygen (^1^O_2_).

PDT is a light-based therapy that uses exposure to light of a specific wavelength to target cancer cells that have picked up a photosensitizer (PS) [152]. The PS is a molecule that, when excited by a specific wavelength of light, produces ROS that kills tumor cells [153]. There are two types of PSs that are used in PDT: type 1 and type 2. Type 1 PSs utilize the electrons and protons they create when excited to generate ROS such as superoxide anions (O_2_^−^) and hydroxyl radical (•OH). Type 2 PSs use the energy they create when excited and O_2_ to produce ROS such as singlet oxygen (^1^O_2_) [154]. However, type 2 PSs face a challenge due to their heavy dependence on ^1^O_2_ to create ROS, since tumor environments are generally hypoxic [155]. Although type 1 PSs are also ^1^O_2_ dependent, they are not completely dependent on ^1^O_2_ and can circulate ^1^O_2_ for repeated use through a series of mechanisms. First, the excited type 1 PS converts the triplet molecular oxygen (^3^O_2_) into O_2_^−^ [156]. Then, the O_2_^−^ is converted into H_2_O_2_ produced through a reaction catalyzed by superoxide dismutase [157]. After H_2_O_2_ is produced, it can be used in a two-step process called the Haber–Weiss reaction for •OH production. The first step is the reaction between ferric iron (Fe^3+^) and O_2_^−^ to produce ferrous iron (Fe^2+^) and ^1^O_2_. The second step, also known as the Fenton reaction, uses Fe^2+^ and H_2_O_2_ to produce •OH and Fe^3+^ for further Haber–Weiss reactions. Additionally, the ^1^O_2_ produced by the first step can be recycled to produce more O_2_^−^, highlighting how type 1 PSs can overcome ^1^O_2_ deficiency in the hypoxic tumor microenvironment [158] (Figure 4).

Since current research in PDT mainly uses type 2 PSs, it is of immense importance to overcome the hypoxic tumor microenvironment [159]. A possible solution to this is using QDs that have been functionalized to deliver PSs as well as molecules that promote ^1^O_2_ synthesis. One study used CDs as delivery agents of the photosensitizer Chlorin e6 (Ce6) for MRI-guided PDT of mice carrying T41 breast cancer tumors [160]. However, these CDs were modified to carry Mn_3_O_4_, Ce6, and PEI since researchers wanted to use the CDs as therapeutic agents as well as diagnostic tools. After being exposed to red light at 660 nm, Ce6 can start generating ROS within the cancer cells, triggering cell death [161,162]. However, the use of Ce6, a type 2 PS, depends on the availability of O_2_, which is usually scarce in cancer cells. To combat this issue, Mn_3_O_4_ is added to the CDs. Mn_3_O_4_ reacts with the abundance of H_2_O_2_ to produce MnO_2_ and O_2_. MnO_2_ also reacts with H_2_O_2_ to produce more O_2_, ensuring that Ce6 has enough O_2_ to generate ROS [160].

Another issue associated with PDT is aggregation-caused quenching (ACQ), which is a decrease in the fluorescence intensity and ROS production of excited PSs due to their largely hydrophobic nature and tendency to accumulate together in hydrophilic biological models [163]. Specifically, PSs tend to accumulate together, since many PSs contain aromatic rings, triggering an effect known as π-π stacking. This is when the pi bonds of aromatic rings are tugged together and bond, causing the PSs to accumulate simultaneously and distort their emission [164]. A possible solution to this is to use QDs that have been functionalized to carry molecules that can fluoresce efficiently despite aggregation, which is a phenomenon called aggregation-induced emission (AIE) [165]. One study in mice models with hepatocellular carcinoma used CDs functionalized with a derivative of tetraphenylene (TPE) called TPETS (a fluorescent TPE core functionalized with sulfur-containing groups) that showed great efficiency in ROS production despite aggregation [166]. Researchers found that CDs functionalized with TPETS could produce more ROS than CDs functionalized with Ce6 when illuminated under light of the same wavelength, highlighting the ability of AIE PSs to overcome ACQ and produce more ROS than traditional PSs. Furthermore, researchers noted that these TPETS-CDs showed greater specificity to light and did not trigger ROS production in dark conditions. The TPETS-CDs only produced ROS in the hepatocellular carcinoma cells when exposed to laser irradiation of 450 nm, suggesting that AIE PSs only generate cytotoxic effects when exposed to a specific light source [166].

Some studies have shown that QDs can be functionalized to be carriers of PS for PDT, which are effective in targeting GB. One study used CdTe QDs bioconjugated with mercaptopropionic acid (MCA) and RGD to target GB in vitro [167]. RGD is a peptide that has a sequence of three amino acids, specifically arginine (R), glycine (G), and aspartic acid (D), and it has been shown to have affinity to the αvβ3 integrin receptor [168]. The αvβ3 integrin receptor is associated with the progression of GB and is present in approximately 60% of GB cases [169]. Thus, bioconjugation with RGD presents a wonderful opportunity for the targeted delivery of QDs to GB cells both in vitro and in vivo. Additionally, MCA-conjugated QDs have been shown to produce ROS for PDT when exposed to light within the range of the visible region [170]. In the human GB U251 cells in vitro, researchers were able to show that MCA-RGD-CdTe QDs were able to significantly reduce tumor cell survival with exposure to visible light at 632.8 nm and found that tumor cell survival decreased with increased treatment time and QD concentration. Furthermore, the tumor cells treated with bioconjugated QDs did not show a significant decrease in cell survival under dark conditions, affirming how these conjugated QDs only produced a cytotoxic effect under exposure to specific light [167]. One study used graphitic carbon nitride (g-C_3_N_4_) QDs as agents for PDT against GB in vitro. However, what makes this study so unique is that the QDs are not conjugated with a PS [171]. Normally, the PS acts as the ROS producer, but in the case of g-C_3_N_4_ QDs, the QDs have a narrow bandgap, meaning that they can absorb less light and still reach an excited state [171]. When excited, g-C_3_N_4_ QDs show great efficacy as both electron acceptors and donors, which is the crucial ability needed to be a PS and generate ROS [171]. Furthermore, within their structure, g-C_3_N_4_ QDs contain molecules called tri-substituted-s-triazines (multipurpose nitrogen-containing heterocyclic compounds with a symmetrical 1,3,5-triazine core), which have been shown to contribute to ROS production [172]. Specifically, in rat GB C6 cells, treatment with g-C_3_N_4_ QDs exposed to light at 488 nm, within the visible region, triggered significant tumor cell death via disruption of the mitochondrial membrane potential by production of ROS [171].

Despite the enormous potential of g-C_3_N_4_ QDs, there are not many studies analyzing the effectiveness of g-C_3_N_4_ QDs for PDT in GB. However, there are studies analyzing the use of conjugated g-C_3_N_4_ QDs as agents for PDT of other cancers, as well as simultaneous drug delivery and bioimaging. One study used PEGylated g-C_3_N_4_ QDs conjugated with folic acid and doxorubicin, a chemotherapy drug, for the treatment of HeLa cells in vitro [173]. Folic acid has high specificity for folate receptor alpha and enables specific delivery of the conjugated QD due to the increased activity of folate receptor alpha in a variety of cancers [174]. Researchers noted that treatment with folic acid and doxorubicin-conjugated g-C_3_N_4_ QDs produced significantly greater anti-tumor effects with exposure to light at 425 nm than treatment with just doxorubicin [173]. Another study with conjugated g-C_3_N_4_ QDs combined the QDs with doxorubicin and carbon nanosheets for the treatment of HeLa cells in vivo [175]. Carbon nanosheets are two-dimensional (2D) sheets of carbon-based molecules, usually graphene or graphdiyne molecules, which have shown applications in bioimaging, biosensing, and drug delivery when functionalized with other molecules [176]. The study found decreased tumor volume as well as successful delivery of doxorubicin when the mice were exposed to light at 808 nm [175]. Although g-C_3_N_4_ QDs are usually excited by light in the visible region, this study was able to stimulate PDT with light at 808 nm, within the near-infrared region, due to the conjugation of the g-C_3_N_4_ QDs with carbon nanotubes, which serve as upconversion nanoparticles (UCNPs) [177]. UCNPs are molecules that can absorb light in one region of the electromagnetic spectrum and convert it to a wavelength of light in a different region, typically converting near-infrared (IR) light to either visible or ultraviolet (UV) light [178]. As such, the carbon nanotubes absorbed the near IR light and produced emissions of visible light that the g-C_3_N_4_ QDs could use to stimulate PDT. The use of g-C_3_N_4_ QDs conjugated with different UCNPs for PDT has been evaluated before, and studies have noted successful conversion of near IR light to visible light, yielding better results for PDT [179,180].

In PDT, the light source is crucial as it is the stimulator for the PS to start ROS production. As such, it is crucial that the light source can penetrate through the body and reach the tumor. However, light sources with a wavelength in the UV or visible range can only penetrate between 0.5 mm and 2.5 mm through the skin, inhibiting PDT from reaching maximum effectiveness for tumors embedded deep within the tissue [181]. However, light with a wavelength in the near IR range, approximately between 760 and 1400 nm, can penetrate up to 10 mm, making it ideal for cancers like GB that frequently present with deep-seated tumors [182,183]. One study with a PS sensitive to near IR light used PEI to bind the PS IR820 to graphene oxide QDs. The QDs carrying IR820, the PS, were then PEGylated to carry MnO_2_ and bovine serum albumin, which enables better conjugation, as well as a cell-penetrating peptide for better delivery outcomes. As mentioned previously, MnO_2_ promotes PDT effectiveness in the hypoxic tumor environment by reacting with H_2_O_2_ to generate O_2_ that can be used for ROS production [160]. Researchers created an in vivo mouse model of GB by xenotransplanting nude mice with human U87MG cells, and after introduction of the functionalized QDs, they noted that the QDs were able to cross the BBB and trigger ROS generation when exposed to near IR light at 808 nm [184]. As such, the development of QDs that are excited by near IR light, as well as QDs that are responsive to near IR via conjugation with UCNPs, will yield better results using improved QDs for the PDT of GB. Furthermore, given the low cytotoxicity and high biocompatibility of g-C_3_N_4_ QDs in vivo, there needs to be further development of bioconjugated g-C_3_N_4_ QDs for the PDT of GB in addition to drug delivery and bioimaging [185].

## 7. Toxicity of QDs Following Use in Precision Imaging, Diagnosis, and Treatment

Despite their efficacy in precision imaging and treatment, there have been concerns about the potentially toxic effects of QDs [186]. Furthermore, studies have also shown that QDs may produce strong immunogenic responses depending on dose and composition [187]. However, most claims arguing against the biocompatibility of QDs are restricted to heavy-metal QDs, such as CdTe and CdSe QDs, that have been shown to leak harmful free cadmium ions in some studies [188]. Although the leakage of free Cd ions challenges the biocompatibility of Cd-based QDs, researchers have already made breakthroughs in limiting the leakage of free Cd ions. In one study, researchers found that lone CdTe QDs had cytotoxic effects on a variety of cell lines due to free Cd ion leakage from the core of the CdTe QD. Later, researchers synthesized CdTe QDs that were covered in a shell made of CdS as well as another shell made of ZnS. The two layers of shells covering the core of the CdTe prevented leakage of free Cd ions, and researchers noted no cytotoxic effects in vitro, even with exposure to the highest available concentration of CdTe/CdS/ZnS QDs [189]. Another study covered CdSe QDs with a ZnS shell and PEGylated the coated CdSe QD to bioconjugate it with DNA sequences that would bind with specific complementary DNA sequences in Xenopus embryos in vivo. Researchers found that these modified CdSe showed specific binding to complementary DNA sequences, stability within the embryo, and no measurable toxic effects [190]. A number of in vivo studies were conducted to assess the toxic effects of QDs following their functionalization with different procedures (Table 3).

Another criticism of the efficacy of QDs is their potential to elicit an immunogenic response upon injection. One study in mice dispersed PEGylated CdTe/ZnS QDs in an aerosol solution. Once mice inhaled the QD aerosol solution, researchers were able to track the movement of the QDs through the olfactory tract and into the brain, specifically the olfactory bulb. Upon histological analysis of the olfactory bulb, researchers found increased activity in the microglial cells, possibly suggesting an inflammatory response, but were unable to find any measurable signs of corresponding cytotoxicity [201]. In another study, researchers used CdTe QDs to study the activation of the NOD-like receptor protein 3 (NLRP3) inflammasome in the nematode *C. elegans*, as well as in the hippocampus of mice [202]. The NLRP3 inflammasome is a protein complex associated with the maturation of pro-inflammatory cytokines and pyroptotic cell death [203]. Free Cd ions have been shown to be potent instigators for the activation of the NLRP3 inflammasome [204]. In this study, researchers observed activation of the NLRP3 inflammasome and other inflammatory markers in mice and *C. elegans* after injection of lone CdTe QDs. However, mice and *C. elegans* injected with CdTe/ZnS QDs reported no measurable activation of the NLRP3 inflammasome and other inflammatory markers due to the ZnS shell preventing leakage of free Cd ions [202]. Currently, most of the research into the cytotoxicity and immunogenicity of QDs is focused on heavy-metal QDs that can create toxic effects when lacking the proper shells or ligands for coating that would prevent the leakage of toxic metal ions. The current literature remains limited in its ability to address long-term biodistribution, degradation, and clearance, especially for metal-based systems such as Cd- and In-containing QDs. Many in vivo investigations focus on analyses that span only days or weeks, which capture immediate cytotoxicity, hematological changes, and gross histological alterations but fail to resolve slower processes such as gradual core dissolution, coating instability, and persistent organ accumulation. At the same time, immune system activation, including both innate and adaptive responses, can manifest over extended timeframes after repeated or prolonged exposure, further complicating safety evaluation. Despite these observations, systematic long-term studies incorporating quantitative biodistribution analysis, detailed clearance pathways, immune profiling, and repeat-dose regimens remain scarce, limiting definitive long-term safety conclusions. Consequently, claims of QD biocompatibility should be explicitly confined to the period of study, and the absence of acute toxicity should not be equated with long-term safety. Future work should prioritize standardized chronic in vivo studies with extended timepoints, cumulative exposure models, and comparative evaluation against biodegradable alternatives to more rigorously assess persistence, immune consequences, and delayed toxicity risks associated with metal-based QDs. However, if one were to extrapolate the cytotoxicity observed with heavy-metal QDs to other QDs like CDs, that would be a gross misinterpretation of the existing data, given how the cytotoxic effects of QDs depend heavily on their concentration, chemical composition, and functionalized ligands [205].

## 8. Advancing QDs with AI for Precision Imaging, Diagnosing, and Treating GB

AI has been used with QDs, specifically for the more efficient development of QDs. In one model, researchers used a data-driven neural network that optimized the synthesis of CuInS2/ZnS QDs by analyzing the previous literature on QD synthesis to provide the best modifications for QD synthesis. QD synthesis is an incredibly delicate process with multiple factors like temperature, ligands, and reaction time influencing how the QD is developed and what its optical properties are. Using information from the previous literature on QD synthesis, the neural network was able to run simulations on how the optical properties of CuInS2/ZnS QDs would change with the modification of the aforementioned factors [206]. Another study that used AI for the synthesis of QDs focused on using machine learning models for the optimized yield of CDs. After exposure to the literature on QD synthesis, the machine learning model was given a set of precursor molecules and produced a set of parameters and instructions for CD synthesis from the given precursors. Furthermore, researchers followed the instructions created by the machine learning model and noted a high yield of 79% [207]. This machine learning model for the optimized synthesis of QDs has been replicated in multiple other studies for the development of a broad range of QDs and presents a wonderful opportunity for the efficient use of precursors in QD synthesis as well as a greater yield of functional QDs [208,209,210].

**Figure 5:**
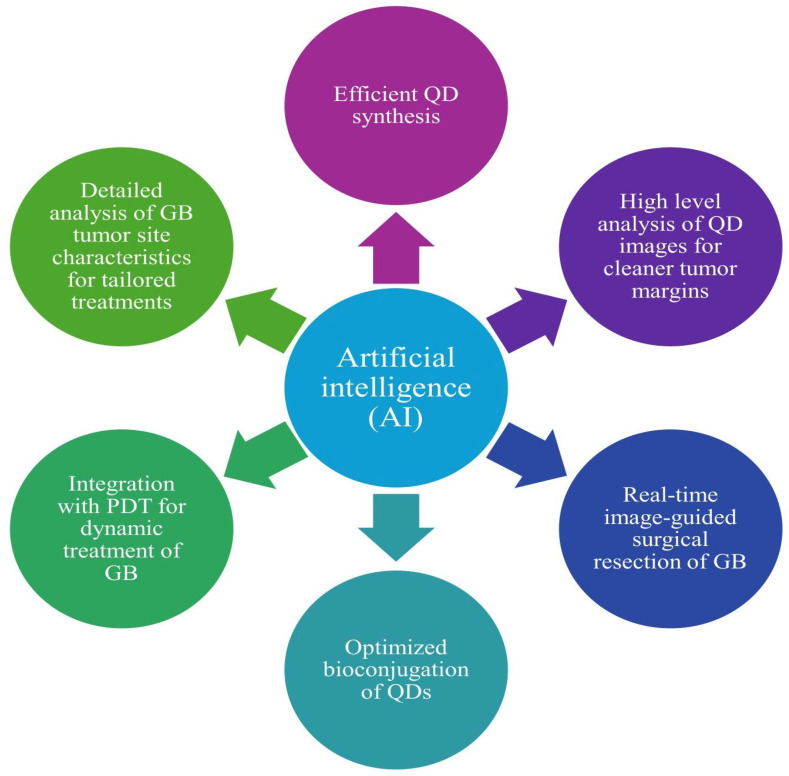
Convergence of QDs and AI for precision imaging, detecting, and treating GB. AI adds an entirely new dimension to the precision imaging, diagnosis, and treatment of GB. Additionally, AI has been shown to have strong compatibility with QDs, highlighting how these two technologies could be used in conjunction with each other to change how GB is diagnosed, treated, and managed. The six examples are only a small fraction of the ongoing research into the relationship between QDs and AI for precision imaging, diagnosing, and treatment of GB.

In addition to QD synthesis, AI has been used in conjunction with QDs for a variety of biomedical applications (Figure 5). In one study, researchers conjugated carboxylated graphene QDs with aptamers that were specific to *Escherichia coli*. The conjugated QDs would bind to *E. coli*, and researchers fed the images of the fluorescent imaging through an AI model that quantifies the number of *E. coli* based on the intensity of the fluorescence from the QDs. Researchers believe that this joint QD-AI model can be useful for the detection of urinary tract infections since the aptamer-conjugated QDs show high bacterial targeting specificity and can be modified to carry aptamers that are specific to different bacteria of interest [211]. In another study, researchers used nitrogen-doped CDs as sensors to differentiate between the presence of warfarin and its metabolites in human serum [212]. They used a machine learning algorithm called linear discrimination analysis to classify warfarin and its metabolites. The algorithm was primed to identify the differences in fluorescence emissions produced by the nitrogen-doped CDs when exposed to warfarin and its metabolites. In human serum, the CD-assisted machine learning model was reportedly more accurate in differentiating between warfarin and its metabolites than the traditional high-performance liquid chromatography analysis [212].

However, given that the use of AI in the biomedical field, especially for QDs with biomedical applications, is still relatively new, there are not many studies analyzing the synergistic effect of AI and QDs for the imaging and treatment of GB. There is one recent study that used Cd-based QDs as contrast agents to perform MRI of GB in humans [213]. After using the Cd-based QDs for MRI, researchers have processed the MRI scans through a convolutional neural network (CNN), which is an AI model commonly used for image analysis of MRI scans [214]. Researchers trained a CNN to identify GB tumor boundaries as well as other prominent features like tumor size, location, and internal heterogeneity, translating the original MRI scans into a set of information that could thoroughly elucidate the characteristics of the tumor. Then, researchers used another AI model, called a generative adversarial network (GAN), which used the information produced by the CNN to create more detailed copies of the original MRI scans. Finally, researchers used the scans produced by this hybrid CNN-GAN model to enable more accurate real-time image-guided radiotherapy [213]. Further studies suggested that adaptation of the QD-AI models could be applied and be instrumental for making further progress in imaging and treating GB (Table 4).

The constructive collaboration of bioconjugated QDs and AI can be realized in the seamless integration of these tools for imaging and treatments of GB, since each technology enhances the other’s capabilities. QDs function as the ultimate data collectors and delivery systems, while AI serves as the intelligent interpreter and orchestrator. For imaging, bioconjugated QDs provide AI models with an unprecedented level of detail and specificity. Instead of just analyzing standard MRI images, AI can be trained on high-resolution fluorescence data from bioconjugated QD-labeled GB tumors. For example, QDs targeting specific GB stem cells within the tumor could provide a map of the most aggressive regions in the tumors. AI algorithms can then use this data to create a detailed, real-time surgical guidance map that highlights these critical areas for resection, something that would be impossible with traditional imaging. Bioconjugated QDs have already been used for targeted molecular imaging and profiling in tumors, offering high specificity and sensitivity [224]. In GB, biofunctionalized carbon and graphene QDs enable precise fluorescent imaging of GB cells and stem cell populations [64]. In GB treatments, QDs carrying drugs can be tracked in real-time with imaging. AI can then analyze this imaging data to monitor the exact location and concentration of the bioconjugated QDs, ensuring that the drug is delivered precisely to the tumor. Furthermore, AI models could use this feedback to dynamically adjust the treatment, for example, by modulating the intensity of a photothermal laser used to activate bioconjugated QDs and kill the GB cells. Graphene QDs have demonstrated dual PTT and PDT capabilities against GB, showing promise for on-demand activation and cell ablation [225]. Such advanced models assist in mechanism-aware drug design by accurately forecasting which conjugated ligands or QD-drug assemblies can cross the BBB [226]. This technology creates a closed-loop system where therapy is continuously monitored and optimized. The constructive collaboration can also be extended to the discovery phase. AI can predict the most effective bioconjugation strategies, designing QDs with the optimal targeting ligands to penetrate the BBB and bind to GB cells. This computational screening process can significantly accelerate the development of new and more effective nanotherapeutics for GB.

## 9. Conclusions and Future Prospects

QDs show great promise in the imaging and treatment of GB. Given their vast range of chemical compositions and optical properties, QDs demonstrate excellent capabilities for targeting GB in various biomedical applications. As of now, the most common strategy involves conjugating QDs with other molecules to enhance their stability, biocompatibility, and ability to target specific tumor cells [64]. Bioconjugated QDs have demonstrated the ability to cross the BBB, enabling their use in both imaging and treatment of GB. Recent advances and applications of QDs have been successful for inducing the receptor-mediated apoptotic cell death in human GB cells [227]. However, GB remains an extremely difficult cancer to image and treat. Challenges such as PsP, ITH, and immunosuppression in the tumor microenvironment present significant obstacles to precision imaging and therapy. Encouragingly, QDs have shown efficacy in overcoming some of these obstacles. Conjugating QDs with distinct biomarkers of GB enables specific targeting of tumor cells, which can help mitigate imaging and treatment issues caused by PsP and ITH. Although this application is promising, further studies are needed to evaluate how effectively QDs address PsP and ITH-related challenges [64]. QDs have also been shown to modulate the immunosuppressive environment of GB. For example, reduced graphene oxide QDs (rGOQDs) loaded with immunomodulatory drugs (R848) and conjugated with anti-PD-L1 antibodies have demonstrated increased T cell activation and reduction in immunosuppressive signaling in GB models. Such targeted immunomodulation offers a compelling strategy to reverse GB-induced immunosuppression [44].

This review primarily focused on using QDs to trigger GB tumor cell death in two ways: conjugating QDs with molecules that trigger apoptosis and the use of QDs in PDT. Surface functionalization of QDs with apoptosis-inducing ligands such as chemotherapeutics, L-Cys, KLA, and doxorubicin enables targeted intracellular delivery and sustained local concentration of these agents within GB cells [49,115,117]. Following cellular uptake, QDs facilitate a variety of apoptotic pathways like mitochondrial dysfunction, disruption of the Bcl-2: Bax balance, cytochrome c release, elevated Ca^2+^ concentration, and DNA damage [228,229,230]. Importantly, QDs can be engineered to cross the BBB and selectively accumulate in GB cells via receptor-mediated targeting, minimizing off-target toxicity. PDT is a promising treatment modality enabled by QDs. Conjugating QDs with photosensitizers allows the production of ROS to trigger tumor cell apoptosis. Although the hypoxic environment of GB tumors typically limits ROS generation, some QD systems can produce oxygen, enhancing PDT efficacy [231]. The gold standard of PDT or any other therapies for GB is the development of the proof-of-principle for inhibition of therapy resistance, including autophagy and induction of apoptosis in GB in vitro and in vivo [232,233,234]. Currently, graphitic carbon nitride QDs (g-C_3_N_4_ QDs) are under investigation for their biocompatibility and innate ROS production capabilities. When conjugated with upconversion nanoparticles (UCNPs), g-C_3_N_4_ QDs can penetrate deep within GB tumors and induce apoptosis through the production of ROS, representing a novel and promising nanotheranostic platform [235,236]. Compared to earlier heavy-metal QDs, which risk cytotoxic metal ion leaching, g-C_3_N_4_ QDs offer strong biocompatibility and lower inherent toxicity [235,236].

The growing application of AI in biomedical research further enhances QD development and application. AI models are being used to optimize QD synthesis and functionalization by exploring high-dimensional reaction spaces and predicting desirable QD properties. They also help interpret real-time QD probe data in complex biological systems [237]. Despite the potential, studies exploring the constructive collaboration between QDs and AI in GB imaging and therapy remain limited, highlighting the need for more research in this area. Successful clinical translation will require coordinated advances across manufacturing, safety evaluation, regulatory strategy, and clinical validation [238]. Comprehensive toxicological profiling and long-term biodistribution studies are essential, particularly to address concerns regarding nanomaterial accumulation, clearance, and chronic toxicity that have historically limited QD advancement into clinical use [239]. Regulatory agencies such as the United States Food and Drug Administration (FDA) currently lack well-defined frameworks tailored to multifunctional hybrid nanomedicines, making early and sustained regulatory engagement critical to clarify product classification, safety standards, and approval pathways for QD-AI combination platforms [240]. Equally important is the prospective validation of AI models on large, multi-institutional clinical imaging and outcome datasets to demonstrate robustness, generalizability, and clinical utility [241]. Finally, sustained collaboration among nanotechnologists, clinicians, and other medical experts will be pivotal in aligning preclinical innovation with translational feasibility, ultimately enabling QD-AI systems to exert meaningful clinical impact in GB management [242]. We hope that the advancement of QDs with click chemistry and their potential constructive collaboration with AI will inspire further research into the rapid integration of these nanotechnologies for improving precision imaging, detecting, and inducing apoptosis in GB in preclinical models, as well as in clinical settings in the near future.

## Figures and Tables

**Table 1:** Bioconjugated QDs are synthesized via click chemistry for biological applications.

Bioconjugated Molecule	QDs	Function of Bioconjugated QDs	Results and Biocompatibility	Reference
Thiomalic acid	Carbon QDs	Fluorescent agents for in vitro and in vivo imaging	The reaction proceeded via a one-pot thiol-ene click approach, and researchers observed a strong yield of the bioconjugated carbon QDs. L929 cells were incubated with the bioconjugated carbon QDs, and researchers noted strong biocompatibility and low cytotoxicity. The fluorescence signals showed that the bioconjugated carbon QDs displayed even distribution in the cytoplasm of the L929 cells.	[65]
Dibenzocyclooctyne (DBCO) and DNA modified with phosphorothioate (PS)	Zn-doped CdTe QDs	In situ labeling of HeLa cells	The DNA was bound on one end by DBCO and by PS on the other end. The -SH end of the PS-bound DNA showed great affinity for the Zn-Doped CdTe QDs, enabling a clickable reaction that does not require a copper catalyst. Research noted a good yield of the bioconjugated QD, as well as no significant cytotoxicity due to the lack of a copper catalyst. The bioconjugated QDs were able to freely enter the HeLa cells and displayed strong fluorescence.	[66]
Arginylglycylaspartic acid (RGD) and DBCO	CdSe/CdS/ZnS core/multishell QDs and ZnCuInSe/ZnS core/shell QDs	Fluorescent agents for in vitro and in vivo tumor imaging	The QDs were coated with a polymer of SPP-N_3_-4VIM, which presented with an azide functionality on the surface. RGD is bound to DBCO, which has alkyne functionality. This enables a clickable reaction that does not need a copper catalyst. RGD shows high affinity for the α_ν_β_3_ integrin proteins expressed on tumor cells, and the bioconjugated QDs displayed high specificity for CT26 cells in vitro. In mice with CT26 cells, the bioconjugated QDs demonstrated prolonged circulation, good biocompatibility, and strong fluorescence in vivo.	[67]
Azide, biotin, Cy5, DBCO, DNA probes, streptavidin	CdSe/ZnS core/shell QDs	Detection of miRNAs in vitro and in vivo	The DNA probes were modified with either azide or DBCO. The probes were then joined via click chemistry without a copper catalyst. The probes were then conjugated with Cy5, a dye for labeling miRNAs, and biotin, which was bound to the QDs that were conjugated with streptavidin. This sensor showed great efficacy in detection of miRNA-155 in HeLa cells and MCF-7 cells. It was also effective at comparing levels of miRNA-155 between control subjects and patients with non-small cell lung cancer.	[68]
5–Norbornene–2–nonanoic acid, tetrazine-PEG, azide	CdSe/CdS core/shell QDs	Labeling of 4T1 cells in vitro	The carboxyl group on 5–norbornene–2–nonanoic acid shows great affinity for metal-based QDs and binds accordingly. Then, the tetrazine-PEG molecules can bind the 5–norbornene–2–nonanoic acid-capped QDs via click chemistry. Azides are added to the other terminus of PEG molecules. The 4T1 cells were treated with DBCO, which could form click reactions with azides. The bioconjugated QDs showed good efficacy in binding to and labeling 4T1 cells that displayed membrane-bound DBCO.	[69]
PEG, azide, alkyne-bearing guangxitoxin-1E	CdSe/CdS core/shell QDs	Imaging of CHO-K1 cells in vitro	The QDs are first coated in PAOA and PMAO, which are amphiphilic copolymers. Then, PEG and azides were added to the surface of the coated QDs. Afterwards, the QDs with the azide functionality were conjugated with the alkyne-bearing guangxitoxin-1E, a potassium channel-specific toxin, in a cooper-catalyzed click reaction. The conjugated QDs showed high affinity for cells with high potassium channel expression.	[70]
Ethylenediamine, citric acid, 2-azidoacetic acid, propargyl alcohol, 8-hydroxy quinoline	Carbon QDs	Inhibiting clinical-resistant bacterial pathogens in vitro	Carbon QDs were treated with ethylenediamine and citric acid to gain amine functionality. The amine-functionalized carbon QDs were then coupled with 2-azidoacetic acid to produce azide functionalized carbon QDs. The azide functionalized QDs were then modified with either propargyl alcohol or 8-hydroxy quinoline via cooper catalyzed click reaction. The QDs conjugated with 8-hydroxy quinoline showed greater effectiveness in inhibiting multi-drug resistant *Staphylococcus aureus* strains compared to the QD conjugated with propargyl alcohol. Furthermore, both conjugated QDs showed no significant cytotoxicity in human cell lines.	[71]

**Table 2:** Both in vitro and in vivo use of QDs for precision imaging and treating GB.

Functionalization	Model	Purpose	Results	Mechanisms	Reference
CDs conjugated with AKRGARSTA	Glio3, Glio 9, and Glio 38 cell lines as well as zebrafish	Imaging	The conjugated CDs exhibited strong biocompatibility and fluorescent imaging in the GB cell lines. These were also able to cross the BBB in the GB zebrafish.	AKRGARSTA showed strong affinity for the p32 receptor, known to be upregulated in GB. This enabled targeted delivery of the conjugated CDs.	[111]
Invitrogen QDot 800 conjugated with EG2-cys	U87MG.EGFRVIII mice	Imaging	The conjugated QDot 800 showed specific binding to GB cells and provided clear imaging free of photobleaching.	EG2-cys showed strong affinity for the EGFRVIII receptor and were widely upregulated in GB. This enabled targeted delivery of the conjugated QDot 800s.	[112]
[^64^Cu]CuInS/ZnS radioactive QDs (RQDs) conjugated with methoxy-PEG-thiol	U87MG mouse xenograft	Imaging	The PEGylated [^64^Cu]CuInS/ZnS RQDs demonstrated functionality with positron emission tomography (PET) scans. PEGylated RQDs had higher levels of uptake in GB cells than glutathione-conjugated RQDs.	PEGylation was key to biocompatibility and tumor uptake. Addition of the [^64^Cu] contributed to radiochemical stability and was key to the conjugated QDs’ PET functionality.	[113]
QDs conjugated with IL-13	GB stem cells	Imaging	The conjugated QDs, composition not specified, were able to bind GB stem cells as well as the exosomes excreted by the GB stem cells.	IL-13 showed strong affinity for the IL13Rα2 receptor and were found to be upregulated in GB. This enabled targeted delivery of the conjugated QDs.	[114]
Ag-In-S ternary QDs conjugated with carboxymethylcellulose (CMC), L-cysteine, and mitochondrial-targeting-peptide (KLA)	U87MG cells grown on chick chorioallantoic membrane	Imaging and treating	The conjugated ternary QDs showed greater efficacy in killing GB cells than treatment with doxorubicin. The conjugated ternary QDs were also effective in reducing tumor angiogenesis. The conjugated ternary QDs retained strong imaging properties.	The CMC helped stabilize the ternary QD for conjugation with L-Cys and KLA. L-Cys functions as a cell-penetrating peptide and shows anti-tumor effects. Once in the tumor cells, KLA disrupted mitochondrial membrane potential, triggering apoptosis.	[115]
CdSeTe/ZnS QDs conjugated with folic acid	U87MG mice	Imaging	The conjugated QDs were delivered through the intrathecal space and showed good efficacy in reaching the tumor site for clear imaging.	Folic acid showed strong affinity for the folate receptor α and was reported to be upregulated in GB. This enabled targeted delivery of the conjugated CdSeTe/ZnS QDs.	[116]
Carboxylated graphene QDs used in coordination with doxorubicin	U87MG cells	Imaging and treating	The carboxylated graphene QDs demonstrated strong membrane permeability as well as specificity for the U87MG cells. When used in coordination with doxorubicin, there was a decrease in tumor cell viability and no reported cytotoxicity to neuronal cells.	It was conjectured that the van der Waals interactions between the carboxyl groups on the conjugated QD and the cell membrane of the U87MG cells were key to increasing the membrane permeability of the graphene QDs.	[117]
CuInS_2_/ZnS QDs conjugated with Gd^3+^ functionalized bovine serum albumin (BSA) protein and anti-CD133 monoclonal antibody	Mice injected with human GB stem cell SU2 line	Imaging	The conjugated CuInS_2_/ZnS QDs showed functionality with magnetic resonance imaging (MRI). The conjugated QD also showed high specificity for targeting the GB stem cells. Analysis of the liver and kidneys presented no measurable signs of cytotoxicity.	The Gd^3+^ functionalized BSA was conjugated to CuInS_2_/ZnS QDs to make the resulting conjugated QDs hydrophilic. Furthermore, the Gd^3+^ functionalized BSA was key for the conjugated QDs’ MRI functionality. The anti-CD133 monoclonal antibody enabled high targeting specificity since the glycoprotein CD133 was upregulated in GB.	[118]
CDs synthesized from L-aspartic acid (L-Asp)	C6 mice	Imaging	The CDs emitted higher fluorescence in the C6 cell site compared to the healthy brain tissue, suggesting high specificity for imaging GB cells. Furthermore, the CDs were able to reach the tumor site following injection in the tail vein, indicating good efficiency in crossing the BBB.	It was proposed that CDs that were synthesized from L-Asp containing functional groups would be picked up by the GLUT-1 and ACT2 transporters, the two transporters responsible for transport across the BBB. Furthermore, L-Asp is a component of RGDs, peptides that show affinity for the α_v_β_3_ integrin receptor. The α_v_β_3_ integrin receptor is upregulated in GB, explaining the high targeting specificity of these CDs.	[119]
Magnetic iron oxide nanoparticles (MIONs) and InP/ZnS QDs loaded into niosomes conjugated with transferrin	U87MG cells	Imaging	The conjugated InP/ZnS QDs showed functionality with MRI. The conjugated QD also showed high specificity for targeting the GB cells.	The MIONSs were conjugated to the QDs to provide MRI functionality. The niosomes (non-ionic surfactant-based vesicles) served as loading molecules to carry transferrin. The transferrin receptor was upregulated in GB, which highlighted the high targeting specificity of these QDs.	[120]
ZnCdSe/ZnS QDs conjugated with c(RGDyk)-poloxamer 188 and bound to microbubbles	Rats injected with C6 cells	Image-guided surgical resection	The conjugated QDs were able to cross the BBB and reach the C6 cells, showing high targeting specificity for the C6 cells. Researchers also used fluorescence imaging from the QDs for image-guided surgical resection and reported clean margins.	c(RGDyk) is an RGD peptide that shows affinity for the α_v_β_3_ integrin receptor. This allowed for specific imaging of the tumor cells. Poloxamer 188 is a derivative of PEG and helps c(RGDyk) bind to the QDs. Microbubbles are transport molecules that demonstrate efficacy in crossing the BBB and enable specific delivery of the conjugated QDs.	[121]
CdTe QDs conjugated with anti-glial fibrillar acidic protein (anti-GFAP)	Mice injected with U87MG cells	Imaging	The conjugated QDs were excellent imaging agents and provided clear fluorescence. The conjugated QDs also showed highly specific targeting of the tumor cells.	Glial fibrillary acidic protein (GFAP) is a well-known biomarker of GB. Anti-GFAP is an antibody that binds GFAP, explaining the high tumor specificity of the bioconjugated QDs.	[122]
CDs conjugated with folic acid and lanthanum (La)	U251 cells	Treating	The conjugated QDs were effective at inhibiting the growth of the U251 cells. Researchers observed a dose-dependent cytotoxic effect on the tumor cells as well as minimal cytotoxic effects on HEK 293 and human umbilical vein endothelial cells (HUVECs).	La has anti-tumor effects, and it also has a tendency to accumulate in GB cells. Folic acid shows a strong affinity for the folate receptor α, which is upregulated in GB. This enabled targeted delivery of the bioconjugated CDs.	[123]
CDs synthesized from metformin and gallic acid	Mice injected with U251 cells	Treating	The CDs showed good capability of crossing the BBB. Researchers reported an increase in tumor death after injection with the CDs. The CDs showed good renal clearance and a lack of renal cytotoxic effects.	The CDs were able to cross the BBB due to their small size, only 2 nm. Tumor cell death was caused by the CDs triggering ferroptosis, a type of cell death caused by the buildup of lipid peroxides and ROS from iron metabolism. The CDs interfere with the function of phospholipid phosphatase 4 (PLPP4), which causes suppression of ferroptosis. Furthermore, the CDs accumulated in the mitochondria, causing morphological changes indicative of ferroptosis.	[124]

**Table 3:** Various in vivo studies conducted for testing toxicity of QDs.

Functionalization	Model	Results	Reference
CDs functionalized with nitric groups	C57BL/6 mice	There was a decrease in body weight, and 4 out of the 7 mice passed away. However, of the mice that passed away, there were no signs of gross toxicity. Furthermore, histopathological analysis of the surviving mice showed no evidence for toxicity.	[191]
CdTe QDs functionalized with PEG	Kunming mice	In mice exposed to CdTe QDs without PEG functionalization, researchers noted oxidative stress in the liver and kidneys. However, mice exposed to CdTe QDs with PEG functionalization showed no oxidative stress in the liver and kidneys, as well as a lack of toxic CD accumulation.	[192]
ZnO QDs	Zebrafish	At concentrations of 2 μg/L of ZnO QDs, the zebrafish embryos displayed no signs of toxicity from the QDs. However, after exposure to concentrations greater than 200 μg/L of ZnO QDs, zebrafish embryos displayed signs of significant toxicity.	[193]
CDs functionalized with carbonyl, hydroxyl, and carboxyl groups	*C. elegans* and BALB/c mice	The CDs showed good biodistribution and a lack of negative effects in both models. Analysis of the dissected stomach and intestinal tissue of the BALB/c mice showed no signs of toxicity.	[194]
InP/ZnS QDs functionalized with either hydroxyl, amino, or carboxyl groups	BALB/c mice	Mice exposed to 2.5 mg of QDs per kg of body weight showed no adverse response to any of the three functionalized InP/ZnS QDs. Mice exposed to 25 mg of QDs per kg of body weight showed no effects on kidney function. However, at this concentration, the InP/ZnS QD functionalized with hydroxyl groups displayed a significant impact on liver function.	[195]
CDs functionalized with hydroxyl and carbonyl groups	Zebrafish	After exposure to the CDs, the zebrafish embryos displayed no significant signs of malformation or morphological change. Furthermore, mortality rates were below 5%.	[196]
Pluronic-encapsulated silicon QDs	Mice and Rhesus macaques	Even after injection of 200 mg of silicon QDs per kg of body weight, both the mice and Rhesus macaques exhibited no significant signs of toxicity over a 3-month period. Researchers observed an elevated presence of silicon within the liver, spleen, and kidneys of the mice. However, this effect was not noticed within the Rhesus macaques.	[197]
CDs functionalized with hydroxyl and carbonyl groups	C57BL/6 mice	At concentrations below 100 μg/mL, the CDs had no immediate negative effects. Furthermore, after 2 weeks, researchers noted no damage to any of the major organs. However, at a concentration of 1 mg/mL, the CDs triggered an immune response and liver damage after 2 weeks.	[198]
Graphene QDs functionalized with carboxyl groups	SKH1 (euthymic, immunocompetent, and hairless) mice	At doses of 5 mg and 10 mg per kg of body weight, researchers were unable to find any signs of toxicity from hematological analysis. Furthermore, after 22 days, analysis of the liver, kidneys, spleen, heart, and lungs found no signs of inflammation or toxicity.	[199]
CdSe QDs coated with a shell of chitosan	BALB/c mice	After injection of the coated CdSe QDs, researchers were unable to find signs of toxicity or physiological changes in the mice for the following 30 days. However, postmortem analysis of liver tissue indicated elevated accumulation of the QDs within liver tissue.	[200]

**Table 4:** Various AI methodologies and convergence with QDs for imaging and treatment of GB.

AI Methodology	Representative Algorithms/Architectures	Training Data Inputs	Proposed Role of QDs	Proposed Applications	Improvements/Outcomes	References
Supervised deep learning	CNNs (U-Net, ResNet, DenseNet, and Visual Geometry Group or VGG)	Multimodal MRI (T1, T2, and FLAIR), QD fluorescence images, voxel-level tumor annotations, and histopathology	QDs provide tumor-specific fluorescence contrast and molecular targeting	Tumor segmentation, surgical planning, and image analysis	Dice similarity coefficient (DSC) is consistently greater than 0.90 with improved tumor boundary detection	[215]
Generative deep learning	GANs (Pix2Pix, CycleGAN)	Paired/unpaired MRI–fluorescence datasets and radiomic features	QDs enhance molecular contrast for high-resolution image synthesis	Radiotherapy guidance, image reconstruction, and enhancement	Greater accuracy in discriminating between PsP and true GB progression than CNN models, and reconstructed images reached a structural similarity index measure (SSIM) of 0.883	[216,217]
Classical machine learning	Linear Discriminant Analysis (LDA), Support Vector Machine (SVM), Random Forest, and k-Nearest Neighbors (k-NN)	QD emission spectra, fluorescence intensity ratios, and lifetime data	QDs function as sensitive fluorescent sensors responsive to molecular differences	Biosensing, molecular discrimination, and fluorescence classification	LDA and SVM differentiated between grade 3 and grade 4 GB with an area under the receiver operating characteristic curve (AUC) of 0.999 and 1, respectively	[218]
Reinforcement learning	Q-learning, Deep Q-Networks (DQN), Policy Gradient Methods	Longitudinal imaging data, QD biodistribution, and tumor response metrics	QDs for analyzing tumor characteristics and supporting treatment	Personalized therapy, adaptive treatment, and treatment optimization	Development of a stimulated tumor microenvironment that includes cytokines, drugs, signaling pathways, and tumor-associated macrophages; obtained an AUC of 0.708 in estimating the survival outcomes of patients with GB	[219,220]
Bayesian optimization	Gaussian Process Regression, Bayesian Search Algorithms	Treatment parameters, imaging feedback, and patient-specific constraints	QDs provide continuous feedback on the tumor site and imaging	Treatment personalization and tumor segmentation	DSC of 0.901 and 0.931 when identifying the enhancing tumor and non-enhancing central necrosis regions of GB in MRI scans	[221]
Graph Neural Networks (GNNs)	Graph Convolutional Networks (GCN), Message Passing Neural Networks	QD size, charge, ligand chemistry, biological performance datasets, and BBB permeability data	QDs are computationally screened prior to synthesis, and ligand-core is modeled for BBB penetration prediction	Drug delivery and nanotherapeutic design	AUC of 0.947 and 0.9212 when identifying compounds as BBB permeable or impermeable, respectively; new GCN models can go beyond prediction and identify key structural elements relevant to BBB permeability	[222,223]

## Data Availability

No new data were created or analyzed in this study. Data sharing is not applicable to this article.
